# Dialysis Recovery Time—Can We Do Better?

**DOI:** 10.34067/KID.0000000000000533

**Published:** 2024-09-26

**Authors:** Peter G. Kerr

**Affiliations:** Monash Health and Monash University, Clayton, Victoria, Australia

**Keywords:** chronic dialysis, dialysis, hemodialysis

There is no doubt that dialysis is lifesaving for people with ESKD. However, the dialysis community remains challenged by the relatively poor mortality outcomes for these people, with mortality rates exceeding those of many malignancies. Furthermore, the morbidity of the dialysis procedure and the symptom burden experienced by patients add to the challenge, all of which have come into greater focus in clinical investigations in recent years. As underscored in the Standardized Outcomes in Nephrology—Hemodialysis (SONG-HD) process, patients on chronic hemodialysis are predominantly burdened by particular symptoms: fatigue, often pronounced after hemodialysis; muscle cramps; and itching.^1^ In this edition of *Kidney360*, Aoun *et al.* explicitly examined the symptom complex of dialysis recovery time (DRT) and fatigue.^2^

DRT is the duration of postdialysis general unwellness that many patients experience, often described as a washed out feeling. In the current study, 15% of patients reported this sensation lasting for more than 12 hours.^2^ If prolonged, this symptom can significantly affect quality of life, leaving patients with very little well time between hemodialysis treatments.

Aoun *et al.* proceeded to examine factors associated with longer DRT and fatigue.^2^ The setting was a group of French dialysis units, involving 536 patients on chronic hemodialysis. DRT was assessed over six consecutive dialysis sessions, whereas overall fatigue was assessed at one time point using the SONG-HD validated fatigue scale.^3^ The cohort displayed good variation in many dialysis parameters that could potentially influence DRT—such as hemodialysis frequency: two times (16%) versus three times (82%) or more than three times (2%) per week, high-flux hemodialysis (37%) versus hemodiafiltration (HDF, 55%) versus hemodialysis with medium cutoff dialyzer membranes (8%)—thus allowing for potential associations to be explored; however, there was a lack of variation in other parameters, such as prescribed blood flow rate and treatment session duration. On the whole, the cohort had high levels of urea clearance (mean eKt/V 1.5) and relatively small intradialytic body weight loss (mean body weight loss 2.5% per hemodialysis session). Although the patient age and gender distribution of the patients (mean age 68.1 years, 61% male) were similar to those seen in other countries, there were fewer participants with diabetes (33%) than might be expected.

Overall, the median DRT was 140 minutes (interquartile range, 45–440), which was better than expected compared with previous reports. Prolonged DRT, defined as a DRT > 12 hours, occurred in 14.9% of patients. Similarly, the SONG-HD fatigue score was lower than in previous studies. Because both measures were better than expected, it may have been more challenging to find associations, potentially necessitating larger patient numbers. The investigators then assessed for associations between dialysis parameters and prolonged DRT, reporting interesting findings. Of the dialysis parameters interrogated, only the intradialytic change (decrease) in serum sodium and reduced hemodialysis frequency (two versus three or more frequent hemodialysis sessions per week) were associated with prolonged DRT. Although this was not a clinical trial offering definitive proof, the observations do pique some interest. There has been a worldwide interest in using lower dialysate sodium concentrations as a means of trying to limit interdialytic weight gains in hemodialysis patients. In turn, this would allow lower ultrafiltration rates, which have been shown to be associated with mortality.^4^ The evidence for this is limited; one small New Zealand randomized clinical trial did not to show a benefit of lowering dialysate sodium in terms of left ventricular hypertrophy.^5^ In addition, a recent observational cohort study involving more than 68,000 patients suggested a higher mortality if dialysate sodium concentrations of 138 mmol/L or less are used.^6^ Although a lower dialysate sodium concentration may diminish interdialytic weight gains as shown in some studies, it may increase the tendency to intradialytic hypotension with poorer cardiovascular tolerance of the dialysis ultrafiltration, although this was not shown in all studies. In all, this should make the dialysis community wary of low dialysate sodium concentrations. Large randomized trials, such as RESOLVE, are needed to clarify this point.

Two hemodialysis sessions per week, as an approach to easing patients into dialysis when they first commence dialysis, that is, incremental hemodialysis, has also gained popularity,^7^ although without randomized trial evidence to support its use. Importantly, two hemodialysis sessions per week is also being employed for sicker patients nearing end of life as a form of decremental hemodialysis. Indeed, some 16% of the cohort were treated with two hemodialysis sessions per week as part of either a decremental and incremental hemodialysis approach. Interestingly, treatment with two hemodialysis sessions per week was independently associated with a longer DRT, despite these patients having smaller ultrafiltration volumes. Statistical analyses were not conducted by dialysis indication for the small subset of patients on two hemodialysis sessions per week, so it is uncertain whether longer DRT was observed in those undergoing incremental hemodialysis, decremental hemodialysis, or both. This distinction is crucial because patients on decremental hemodialysis are typically sicker than those on incremental or conventional hemodialysis. Moreover, other studies showed that incremental hemodialysis is associated with shorter DRT.^8^ There are a number of reasons why incremental hemodialysis has been promoted in recent years, but one of them has been the proposal that it is associated with improved quality of life for the patients. Perhaps like the dialysate sodium trend, the hard evidence for this is thin and indeed lacking in the small clinical trials conducted so far. The French data raise concerns about one aspect of quality of life with this approach. Although clinical trials are again needed to clarify this, recruiting patients to incremental hemodialysis trials has proven difficult, with <15% of patients commencing hemodialysis being able to be recruited.^9^

HDF has become a popular dialysis modality in Europe. However, there remains some skepticism as to its benefits, despite several clinical trials showing survival benefit, with the most recent being the CONVINCE trial.^10^ Indeed, clinical trials comparing hemodialysis with HDF had some caveats, leaving some uncertainties about the added benefits with this modality. Unexpectedly, the study by Aoun *et al.* reported longer DRT in elderly patients managed with HDF. However, this cohort subset (elderly patients on HDF) was small, and these results only add to the controversies surrounding HDF.

Equally important are the factors that did not associate with prolonged DRT and fatigue. Parameters such as eKt/V, ultrafiltration rates (and interdialytic weight gain), BP, and change in BP did not show an association with prolonged DRT or increased fatigue. Similarly, except in the elderly patients, dialysis modality or membrane type failed to associate with DRT. Whether this reflected the sample size or the relative lack of variation of some of these factors remains unclear. On the other hand, hemoglobin (Hb) levels associated with fatigue, although weakly, and at a level considered well within the target range for most Hb management protocols. This information is at odds with older data from clinical trials testing different Hb goals with erythropoietic-stimulating agents but does agree with intuitions about anemia and fatigue.

While the study can be considered hypothesis generating rather than being conclusive, it does emphasize the need for well-conducted clinical trials. Nephrology, and especially dialysis, has proven a difficult landscape for the conduct of clinical trials. The large imposition already of the hemodialysis procedure on the patients' life style and well-being makes recruiting to studies difficult both in terms of patient and investigator preparedness. High mortality and morbidity rates, influenced seemingly by a large number of factors, appear to dampen the predicted responses to therapy and have made interpretation and generalizability difficult. Particularly since the Standardized Outcomes in Nephrology initiative, attention has focused on quality-of-life outcomes. Studies, such as conducted by Aoun *et al.*, are to be applauded, yet remain interesting without being conclusive. Several studies are currently underway which might illuminate the impact on symptom burden and quality of life with lower dialysate sodium concentrations, incremental hemodialysis, and HDF. The nephrology community, patients, and clinicians alike, eagerly await the results.
